# Retrospective study of FAPI imaging in bone metastases: diagnostic utility and therapeutic relevance across cancer types in a single-center real-world cohort

**DOI:** 10.1186/s13550-026-01502-z

**Published:** 2026-08-11

**Authors:** Xinru Li, Xi Jia, Chenzhen Li, Huixing Deng, Tao Tian, Wei Zhao, Xiao Fu, Yuanbo Wang, Yan Liu, Aimin Yang, Bing Jia, Rui Gao

**Affiliations:** 1 Departments of Nuclear Medicine, The First Affiliated Hospital of Xi’an Jiaotong University, No. 277 Yanta West Road, Xi’an, 710061 P.R. China; 2 Medical Isotopes Research Center, Department of Radiation Medicine, School of Basic Medical Sciences, Peking University, Beijing, 100191 P.R. China; 3 Departments of Medical Oncology, The First Affiliated Hospital of Xi’an Jiaotong University, Xi’an, 710061 China; 4 Departments of General Surgery, The First Affiliated Hospital of Xi’an Jiaotong University, Xi’an, 710061 China

**Keywords:** FAPI imaging, [^99m^Tc]Tc-H7ND SPECT/CT, Bone metastases, Diagnostic efficiency

## Abstract

**Background:**

Bone metastases frequently occur in advanced-stage malignancies, contributing to diminished quality of life and unfavorable prognoses. However, conventional imaging modalities for diagnosing bone metastases exhibit inherent limitations. This study aimed to evaluate the diagnostic utility of fibroblast activation protein inhibitor (FAPI) imaging using [^99m^Tc]Tc-H7ND for detecting bone metastases (BM). From June 2021 to September 2023, 564 patients with malignancies underwent FAPI imaging were screened. Eighty patients with confirmed or suspected BM were included. FAPI SPECT/CT was compared with CT, MRI, bone scintigraphy (BS), and [¹⁸F]FDG PET/CT for diagnostic efficacy. The uptake values in different components of BM were assessed, alongside discussions on potential of FAPI imaging in evaluating treatment response.

**Results:**

Eighty patients (median age: 60 years; range: 23–79 years; 48 men) with confirmed or suspected BM were included, of these, 64 were confirmed BM. FAPI SPECT/CT demonstrated superior sensitivity (96.5%) and accuracy (95.9%) compared to CT (59.7%, 67.1%; *p* < 0.001), detecting significantly more lesions than CT (*p* < 0.001) and MRI (*p* = 0.041). FAPI imaging outperformed BS in detecting osteolytic and mixed metastases, identifying 12 and 9 additional lesions, respectively. FAPI uptake differed significantly among BM subtypes, with median SUV_max_ values of 13.41 (osteolytic), 10.78 (mixed), and 2.70 (osteoblastic) (*p* < 0.001). Compared to conventional imaging combinations, FAPI identified new lesions in 46.3% of patients, prompting treatment modifications in 86.5% of cases. For patients who underwent follow-up FAPI imaging, changes in SUV_max_ of BM correlated with RECIST 1.1 criteria.

**Conclusion:**

The findings may enhance diagnostic performance in detecting BM across malignancies, particularly for osteolytic and mixed metastases. Preliminary observations also suggest possible applications in monitoring therapeutic response, though further validation is required to confirm these exploratory findings. This is a retrospective study, utilizing data collected from a registry (registration number: ChiCTR2100048093; date of registration: June 30, 2021).

**Supplementary Information:**

The online version contains supplementary material available at https://doi.org/10.1186/s13550-026-01502-z.

## Background

Bone metastases (BM) are common in advanced-stage malignancies and significantly impact patient quality of life and prognosis [1, 2]. Accurate diagnosis of BM is critical for effective cancer management.

While conventional CT and MRI demonstrate high accuracy in detecting regional BM [3, 4], their utility is limited by the systemic nature of metastatic disease. Early BM often remains undiagnosed until morphological changes occur [5]. Bone scintigraphy (BS) using [^99m^Tc]Tc-labeled diphosphonates has been widely employed for BM detection [6], though its limited specificity reduces clinical utility [7]. Damle NA et al. proposed [^18^F]NaF PET/CT as a potential alternative to [^99m^Tc]Tc-MDP [8]; however, broader adoption requires cost-effectiveness evaluations. Furthermore, monitoring treatment response remains challenging with modalities like CT, BS, and [^18^F]NaF PET/CT, which rely on detecting bone destruction or reactive remodeling [5]. These limitations underscore the need for more precise and cost-effective diagnostic methods.

Since its introduction in 2018, radionuclide-labeled fibroblast activation protein inhibitor (FAPI) imaging has garnered significant attention in nuclear medicine [9]. Cancer-associated fibroblasts (CAFs), key contributors to BM pathogenesis [10–12], have spurred interest in FAP-targeted imaging for BM evaluation. Although preliminary studies suggest a role for FAPI imaging in detecting BM [13], its specific potential in this context remains largely unexplored.

[^99m^Tc]Tc-H7ND is a novel FAP-targeted diagnostic agent with optimized structure for enhanced tumor-to-background ratio. Prior clinical investigations with [^99m^Tc]Tc-H7ND (ChiCTR2100048093, NCT05999214, NCT06443710) confirm the safety and strong FAP affinity, supporting its diagnostic potential. In this retrospective study, we assessed the diagnostic performance of [^99m^Tc]Tc-H7ND in patients with suspected or confirmed BM across various tumor types, comparing it to traditional imaging modalities. We also analyzed FAPI uptake patterns in different BM subtypes and evaluated subsequent changes in treatment regimens prompted by imaging findings.

## Results

### Patient characteristics

**Fig. 1:**
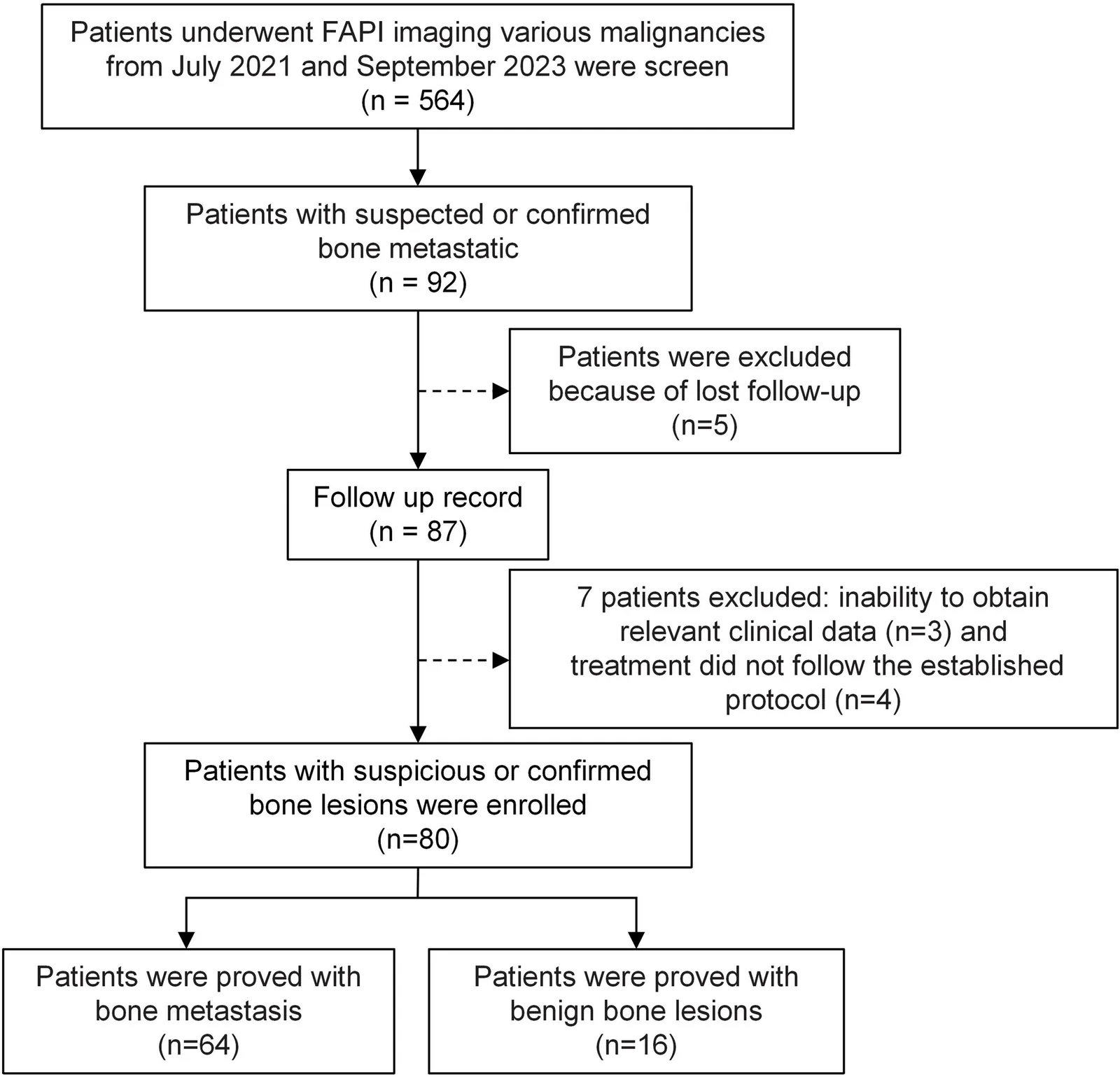
The scheme of the study design

Between July 2021 and September 2023, we retrospectively analyzed 564 consecutive patients with suspected or confirmed malignancies who underwent FAPI imaging at the Department of Nuclear Medicine, First Affiliated Hospital of Xi’an Jiaotong University. Of these, 92 patients were initially screened, with 80 meeting the inclusion criteria and subsequently enrolled in the study. The enrollment flowchart is illustrated in Fig. 1, and detailed patient characteristics and clinical information are summarized in Table 1. No adverse events were reported or observed in any patient following the administration of [^99m^Tc]Tc-H7ND or during the observation period.

**Table 1:** Patients Characteristics

Characteristic		Number
No. of patients		80
Age (years)	Median (range)	60 (23–79)
Sex	Male	48 (60%)
	Female	32 (40%)
Site and pathology of primary disease		
	Lung	55 (68.75%)
	Adenocarcinoma	35
	Squamous carcinoma	12
	Small cell lung cancer	6
	Others	2
	Genitourinary	14 (17.5%)
	Bladder cancer	5
	Prostate cancer	4
	Ovarian cancer	2
	Renal cancer	2
	Ureter cancer	1
	Sarcoma	3 (3.8%)
	Osteosarcoma	1
	Chondrosarcoma	1
	Liposarcoma	1
	Breast cancer	2 (2.5%)
	Pleura mesothelioma	2 (2.5%)
	Others	4 (5.0%)
	Gastric adenocarcinoma	1
	Laryngocarcinoma	1
	Cutaneous squamous cell carcinoma	1
	Thymic carcinoma	1
Clinical status before imaging		
	Treatment-naive	7 (8.8%)
	Resection surgery	3 (3.8%)
	Chemo/radio/target/radio-therapy after surgery	34 (42.5%)
	Systemic chemo/immune/target-therapy	36 (45.0%)
Other imaging		
	CT	73 (91.3%)
	Bone scintigraphy	36 (45.0%)
	MRI	24 (30.0%)
	[^18^F]FDG PET/CT	14 (17.5%)

## Higher diagnostic accuracy with FAPI imaging than traditional ones in BM

Using clinical, imaging, and histopathological reports as the reference standard, BM were confirmed in 80.0% (64/80) patients. For FAPI imaging, the diagnostic sensitivity, specificity, negative predictive value (NPV), positive predictive value (PPV) and accuracy were 96.9% (62/64), 93.8% (15/16), 88.2% (15/17), 98.4% (62/63) and 96.3% (77/80), respectively, yielding a Youden’s index of 0.907. These diagnostic performance metrics are summarized for all modalities in Supplementary Table S1. To ensure comparability, only patients who underwent both FAPI imaging and conventional imaging studies within a 28 days interval were included in the comparative analysis. In the patient-based analysis, FAPI imaging demonstrated superior sensitivity and accuracy compared to CT, with sensitivities of 96.5% vs. 59.7% (*p* < 0.001) and accuracies of 95.9% vs. 67.1% (*p* < 0.001). While FAPI imaging also demonstrated numerically higher sensitivity and accuracy compared to MRI, BS, and [¹⁸F]FDG PET/CT, these differences lacked statistical significance, likely due to limited sample sizes. Detailed comparative data are presented in Table 2.

**Table 2:** A Patient-based Paired Comparison of FAPI imaging with Other Methods for Detection BM

	Basis of analysis	Sensitivity (TPR) 95% CI	Specificity (TNR) 95% CI	Accuracy (ACC) 95% CI	Youden’s index
FAPI vs. CT (*n* = 73)	FAPI	96.5 (55/57)	93.8 (15/16)	95.9 (70/73)	0.903
		87.9–99.4	69.8–99.8	88.5–99.1	
	CT	59.7(34/57)	93.8 (15/16)	67.1 (49/73)	0.535
		45.8–72.4	69.8–99.8	55.1–77.7	
	P value	< 0.001	/	< 0.001	
FAPI vs. MRI (*n* = 24)	FAPI	95.2 (20/21)	100 (3/3)	95.8 (23/24)	0.952
		76.2–99.9	29.2–100	78.9–99.9	
	MRI	76.2(16/21)	100 (3/3)	79.1 (19/24)	0.762
		52.8–91.8	29.2–100	57.9–92.9	
	P value	0.089	/	0.194	
FAPI vs. MDP (*n* = 36)	FAPI	96.7 (29/30)	100.0 (6/6)	97.2 (35/36)	0.967
		82.8-99.96	54.1–100	85.5–99.9	
	MDP	86.6(26/30)	83.3 (5/6)	86.1 (31/36)	0.699
		68.3–95.6	36.5–99.1	70.5–95.3	
	P value	0.355	0.297	0.199	
FAPI vs. FDG (*n* = 14)	FAPI	100 (12/12)	100 (2/2)	100 (14/14)	1.000
		73.5–100	15.8–100	76.8–100	
	FDG	66.7(8/12)	100 (2/2)	71.4 (10/14)	0.667
		34.9–90.1	15.8–100	41.9–91.6	
	P value	0.093	/	0.098	

FAPI: FAPI SPECT/CT; BM: bone metastases; BS, Bone scintigraphy; FDG: [^18^F]FDG PET/CT; TPR: true positive rate; TNR: true negative rate

## FAPI imaging demonstrates superior detection of bone metastases compared to CT, MRI, and ^18^F-FDG PET/CT

**Fig. 2:**
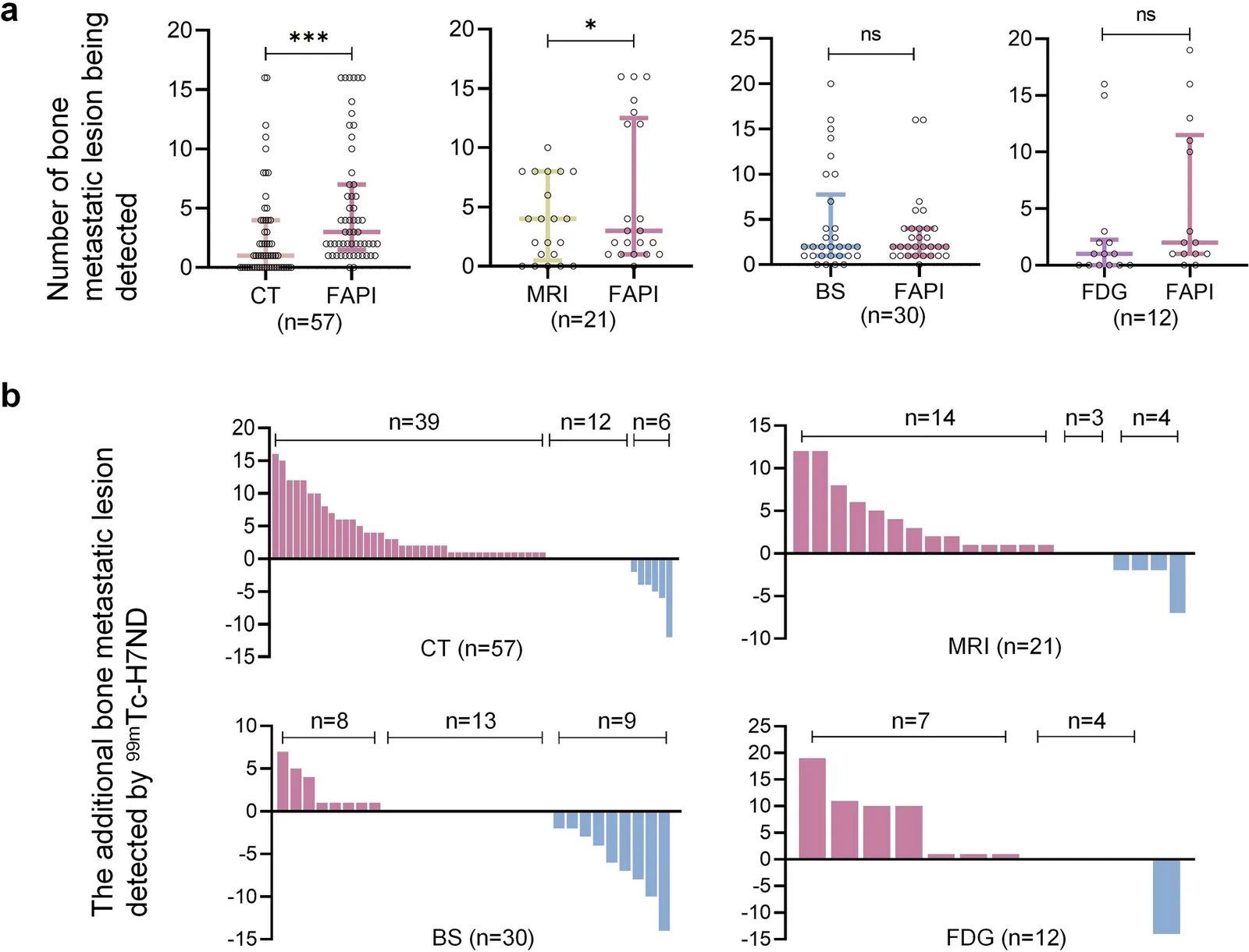
Detection of bone metastatic lesions by various imaging modalities. **a**, Patient-based paired comparison of the number of bone metastases detected by different imaging modalities. ns, none significance; *, *p* < 0.05; ***, *p* < 0.001. **b**, Additional bone lesions detected by FAPI imaging. BS, Bone scintigraphy; FDG, [^18^F]FDG PET/CT

In 64 patients with BM, a total of 404 bone lesions were identified. The mean number of lesions detected per patient was lower for CT, MRI, BS, and [^18^F]FDG PET/CT compared to FAPI imaging, with results of 2.70 ± 0.523 (*n* = 57), 3.81 ± 0.739 (*n* = 21), 4.63 ± 1.023 (*n* = 30), and 3.42 ± 1.654 (*n* = 12), respectively, vs. 5.28 ± 0.666 (*n* = 64) for FAPI imaging. Notably, CT and [^18^F]FDG PET/CT detected significantly fewer lesions than FAPI imaging (*p* < 0.001 and *p* = 0.021, respectively; see Supplementary Fig. S1). To further assess detection efficacy, paired comparisons were performed between FAPI imaging and each modality (Fig. 2a). FAPI imaging detected significantly more lesions than CT (5.12 ± 0.669 vs. 2.70 ± 0.523; *p* < 0.001; *n* = 57) and MRI (6.00 ± 1.324 vs. 3.81 ± 0.739; *p* = 0.041; *n* = 21). While no significant differences were observed between FAPI imaging and BS (3.47 ± 0.694 vs. 4.63 ± 1.023; *p* = 0.201; *n* = 30) or [^18^F]FDG PET/CT (6.67 ± 1.936 vs. 3.42 ± 1.654; *p* = 0.141; *n* = 12). To further illustrate the superior lesion detection capability of FAPI imaging, the differences in lesion detection between modalities per patient-based analysis were quantified (Fig. 2b). FAPI imaging identified more lesions than CT in 68.4% (39/57) of patients, while detecting fewer lesions in only 10.5% (6/57). Similarly, FAPI imaging revealed more BM than MRI and [^18^F]FDG PET/CT in 66.7% (14/21) and 58.3% (7/12) of patients, respectively.

**Fig. 3:**
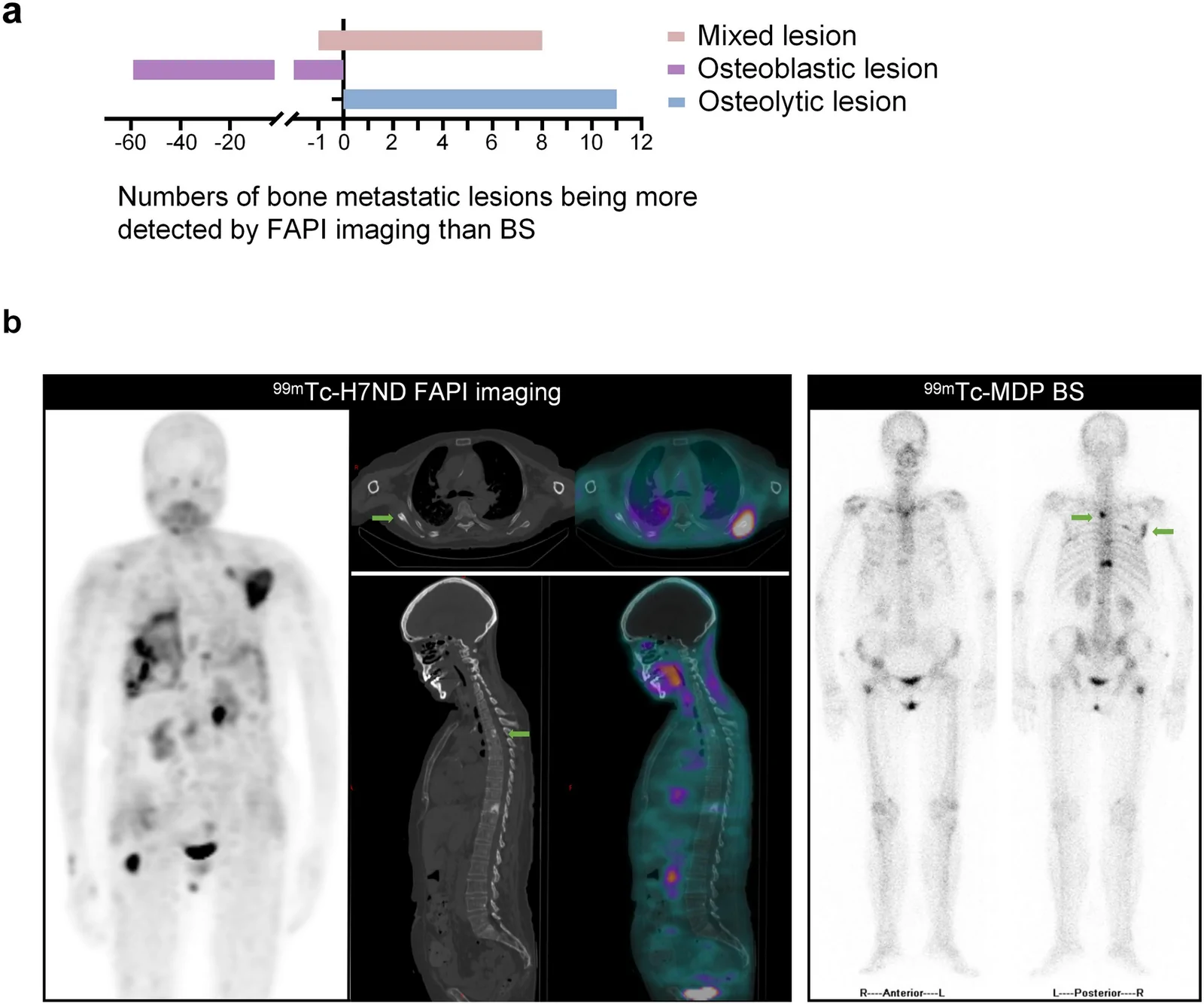
The combination of FAPI and BS complemented each other to detect BM. **a**, Numbers of bone metastatic lesions being more detected by FAPI imaging than BS in different types of bone lesions. **b**, Representative imaging of FAPI and BS in a NSCLC patient with multiple osteoblastic BM. FAPI results were on the left, with 2 representative CT and SPECT/CT fusion figure of scapula lesion (above) and thoracic vertebra lesion (below). Anterior-posterior BS image was on the right. Green arrows indicate bone lesions positive on BS but negative on FAPI imaging

Although the mean number of lesions detected by BS did not significantly differ from that identified through FAPI imaging, BS revealed a greater number of lesions in 30% of patients (9 out of 30) (Fig. 2b). Notably, FAPI imaging surpassed BS in the detection of osteolytic and mixed metastases, identifying 12 and 9 additional lesions, respectively, that were often missed by BS; only one osteolytic lesion was overlooked by FAPI. In contrast, BS was more effective in detecting osteoblastic lesions, identifying 59 more lesions while missing only one (Fig. 3a, b). To further quantify the complementary value of the two modalities, we performed an additional patient-level combined analysis in the 30 patients who underwent both FAPI and BS. FAPI alone achieved a patient-based sensitivity of 96.7% (29/30), while BS alone achieved 86.7% (26/30). When interpreted in combination (positive if either modality was positive), the sensitivity reached 100% (30/30). These data confirm that FAPI and BS are not redundant but rather complementary.

## Lesion-based FAPI uptake value correlates with BM composition

**Fig. 4:**
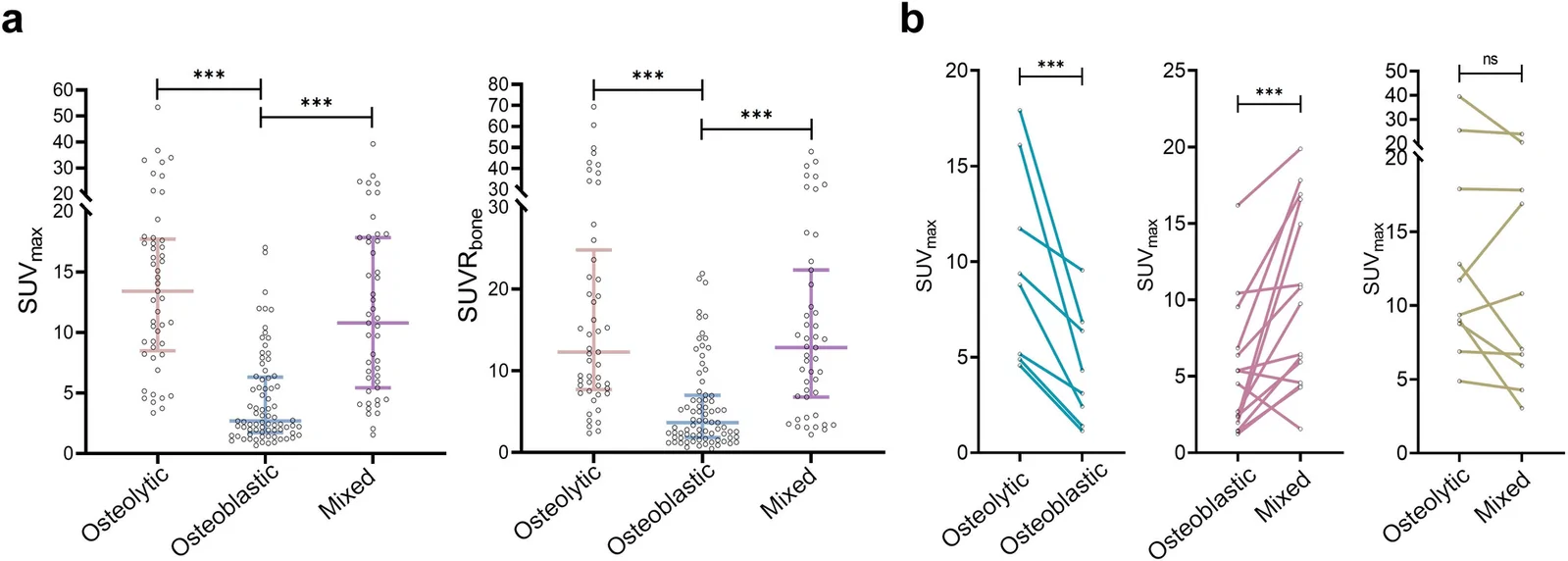
The uptake of FAPI in BM with different types of BM. **a**, Lesion-based analysis of bone uptake of FAPI calculated with SUV_max_ (left) and SUVR_bone_ (right) in osteolytic, osteoblastic and mixed BM. **b**, Paired comparison of bone uptake of FAPI in patients with different types of BM. ns, none significance; ***, *p* < 0.001

A total of 173 bone metastatic lesions were evaluated, revealing a median SUV_max_ of 7.060 (95% CI: 5.43–8.94; interquartile range [IQR]: 3.06–14.29), a median SUV_mean_ of 4.55 (95% CI: 3.51–5.35; IQR: 2.06–8.43), and a median SUVR_bone_ (dividing SUV_max_ of the bone metastatic lesion by the corresponding SUV_mean_ of normal bone) of 7.62 (95% CI: 6.39–9.75; IQR: 3.13–15.37). To assess the uptake of FAPI across different types of BM, separate analyses were conducted based on lesion type. As illustrated in Fig. 4a, significant differences were observed among osteolytic, osteoblastic, and mixed bone metastases (*p* < 0.001). Specifically, osteolytic bone metastases demonstrated higher uptake compared to osteoblastic lesions, with a median SUV_max_ of 13.41 (IQR: 8.50-17.71) versus 2.70 (IQR: 1.74–6.32) (*p* < 0.001). Similar results were noted when comparing mixed bone metastases to osteoblastic lesions, with median SUV_max_ values of 10.78 (IQR: 5.43–17.83) versus 2.70 (IQR: 1.74–6.32) (*p* < 0.001). However, the difference in uptake between osteolytic and mixed bone metastases did not reach statistical significance (*p* = 0.147).

Intra-patient comparisons of BM subtypes (Fig. 4b) further confirmed these trends: osteolytic and mixed BM lesions retained significantly higher FAPI uptake than osteoblastic lesions (*p* = 0.008 and *p* = 0.001, respectively), while no statistically significant difference was observed between osteolytic and mixed lesions (*p* = 0.106).

## FAPI imaging influenced treatment decision making in patients with various tumors

**Fig. 5:**
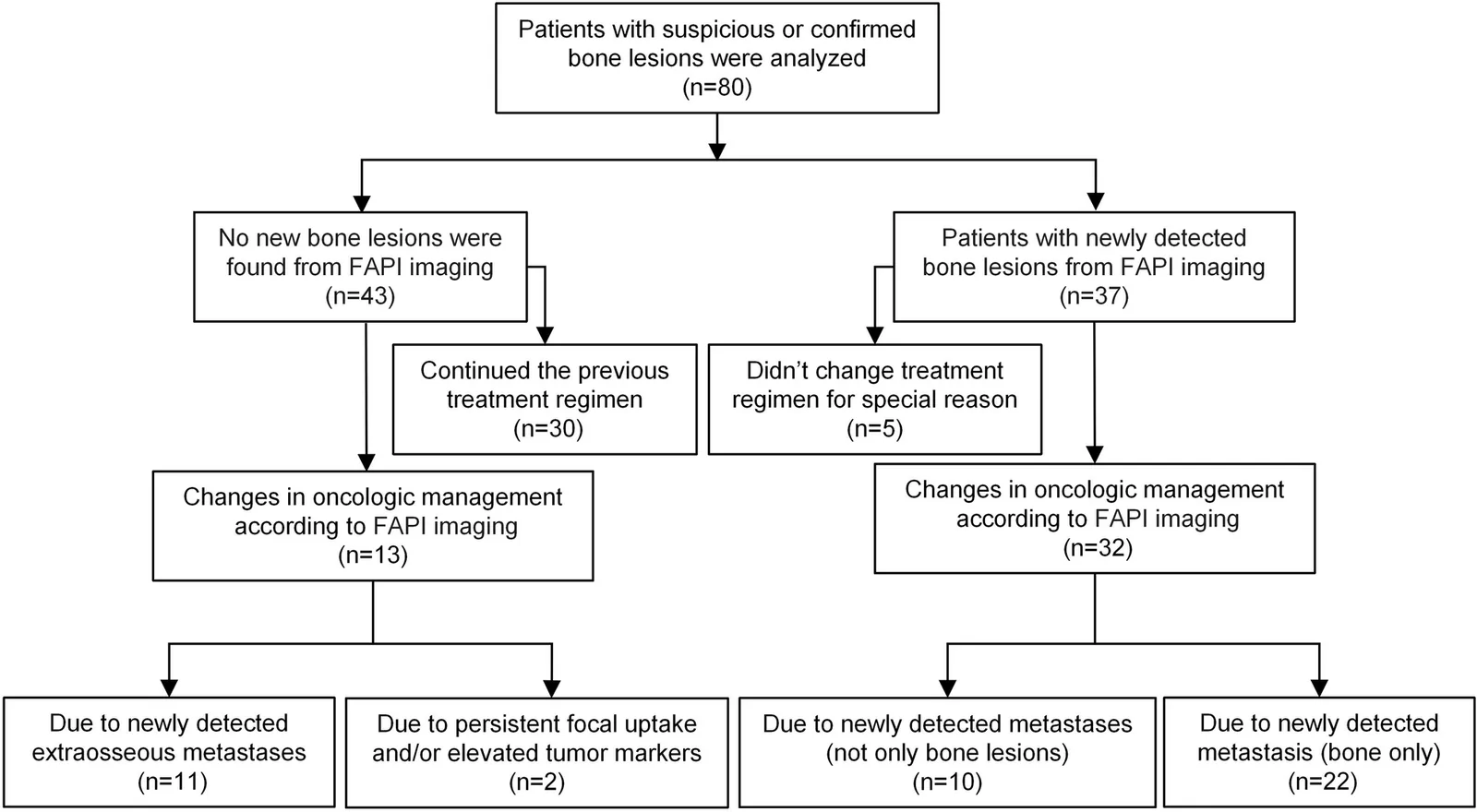
Impact of FAPI imaging on treatment decision-making

Eighty patients with suspicious or confirmed bone lesions were analyzed. FAPI imaging identified newly detected bone lesions in 37 patients (46.3%), of whom 86.5% (32/37) underwent subsequent modifications to their treatment regimen. Among these changes, 59.5% (22/32) were directly attributable to FAPI-guided detection of new BM (Fig. 5).

While the study focused on BM, FAPI imaging also facilitated the identification of primary tumors and extraosseous metastases. In two cases, FAPI imaging not only confirmed BM but also localized previously undiagnosed primary lesions (renal and gastric, respectively), both pathologically validated (Supplementary Fig. S2). Additionally, extra-skeletal metastases were detected via FAPI in 23 patients.

Overall, FAPI imaging contributed to a change in oncologic management for 56.3% of the patients (45 out of 80). This included alterations in systemic treatment regimens for 34 patients, confirmation of the continuation of systemic therapy over surgical or local approaches for 6 patients, synching local treatment with systemic chemotherapy for 2 patients, performing local bone surgery in conjunction with systemic therapy for another 2 patients, and the administration of local radiotherapy alongside targeted therapy for 1 patient.

### Serial FAPI imaging was useful for assessing treatment efficacy of BM

Serial FAPI imaging was performed in eight patients to evaluate treatment response in BM. Changes in SUV_max_ for the five most metabolically active BM lesions were analyzed alongside RECIST 1.1 criteria. As summarized in Table 3 and Supplementary Fig. S3a, three patients classified as having stable disease (SD) by RECIST 1.1 exhibited reductions in SUV_max_. Similarly, three patients categorized as non-partial response/non-progressive disease (nPR/nPD) demonstrated substantial decreases in SUV_max_. In contrast, two patients with progressive disease (PD) displayed significant SUV_max_ increases and developed new metastatic lesions.

**Table 3:** Changes of the Uptake of Bone Metastatic Lesions in Patients Who Underwent reviewed FAPI imaging

No./Sex/Age	Primary tumor	Type of bone metastasis	Newly detected bone metastasis from FAPI imaging	Changes of treatment regimen	Repeated H7ND of delta-SUV_max_	RECIST 1.1 evaluation result	Note
1/M/52	Prostate Cancer	Mixed and osteoblastic	No	Yes	-11.87%	SD	PSA declined and most of the lesions turned into osteoblastic
2/M/66	Lung adenocarcinoma	Mixed and osteoblastic	No	No	-49.6%	SD	Soft tissue lesions decreased in diameter
3/M/60	Lung squamous carcinoma	Mixed and osteolytic	No	No	-24.6%	SD	Decreased osteoclast composition
4/M/63	Bladder cancer	Osteoblastic	Yes	Yes	-60.9%	nPR/nPD	/
5/F/53	Lung adenocarcinoma	Osteoblastic	No	No	-50.7%	nPR/nPD	/
6/M/30	Thymic carcinoma	With all types	Yes	Yes	-78.03%	nPR/nPD	
7/M/55	Small cell lung cancer	Mixed and osteolytic	Yes	Yes	+ 43.9%	PD	More bone metastatic lesions were found
8/F/59	Lung adenocarcinoma	With all types	No	Yes	+ 84.70%	PD	More bone metastatic lesions were found

SD: stable disease; nPR/nPD: non-partial response/non-progressive disease; PD: progressive disease

Notably, one patient with newly detected bone lesions on initial FAPI imaging did not receive immediate treatment adjustments. However, follow-up imaging after two months revealed additional bone metastases and new hepatic lesions (Supplementary Fig. S3b). Lesion-specific SUV_max_ changes are detailed in Supplementary Table S2.

## Discussion

In this study, we demonstrated the exceptional diagnostic efficacy of [^99m^Tc]Tc-H7ND-based FAPI imaging in detecting bone metastases compared to other imaging modalities. A key finding is the significantly higher uptake of FAPI in osteolytic and mixed BM compared to osteoblastic lesions, highlighting the modality’s sensitivity to varying metastatic phenotypes. Furthermore, the integration of bone scintigraphy (BS) with FAPI imaging was shown to markedly enhance diagnostic accuracy, underscoring the complementary nature of these techniques. Moreover, FAPI imaging’s reliable performance supports its potential as a valuable tool for clinical decision-making and a supplement to traditional therapeutic evaluation systems. By capturing molecular changes beyond morphological alterations, this approach offers a more comprehensive assessment of treatment response in BM. This comprehensive strategy establishes a valuable reference point for evaluating therapeutic efficacy in BM, paving the way for more personalized and precise management of metastatic disease.

BM has long posed a clinical challenge in diagnostic imaging and oncology. BS using [^99m^Tc]Tc-MDP remains the recommended standard for BM detection and is routinely paired with CT or MRI [14]. However, current diagnostic approaches exhibit significant limitations in identifying BM with complex pathological changes, particularly in cases involving osteolytic lesions characterized by bone destruction and resorption [5]. [^99m^Tc]Tc-MDP is a widely used bone imaging agent for detecting BM due to its chemisorption to hydroxyapatite crystals [15], increasing uptake in areas of high osteogenic activity [7]. Nevertheless, lesions dominated by tumor-mediated bone replacement or compromised vascular supply often demonstrate reduced radiotracer uptake, hindering accurate detection [8]. While [¹⁸F]FDG or [¹⁸F]NaF PET/CT may improve diagnostic precision for BM, these modalities are not routinely recommended in clinical practice [16]. Emerging molecular imaging agents such as prostate-specific membrane antigen (PSMA) show promise for prostate cancer BM diagnosis [17], though systematic evaluation across diverse malignancies remains lacking. Our findings demonstrate that FAPI imaging, a broad-spectrum tumor-targeting modality, surpasses CT, MRI, and [¹⁸F]FDG PET/CT in detecting BM lesions. FAPI imaging achieved superior diagnostic accuracy, detected additional lesions, and effectively compensated for the limitations of BS in visualizing osteolytic metastases across multiple cancer types. This positions FAPI as a more versatile tool for BM diagnosis. Furthermore, combining BS with FAPI imaging may enable comprehensive diagnostic evaluation of BM, warranting further investigation into hybrid approaches.

The strong uptake of FAPI in bone marrow lesions reflects a high abundance of cancer-associated fibroblasts (CAFs), which play a critical role in modifying the bone matrix and facilitating metastatic progression [11]. CAFs interact with tumor cells to establish a metastatic niche, promoting bone metastasis through the secretion of growth factors, cytokines, and chemokines that enhance cancer cell proliferation, survival, and migration [18–20]. FAP, a key marker of CAFs, contributes to BM progression and osteoclastic activity by modulating matrix metalloproteinases (MMPs) and transforming growth factor-beta (TGF-β), thereby increasing bone resorption [21, 22]. This mechanism explains our observation that FAPI uptake was significantly higher in osteolytic lesions (median SUV_max_ 13.41) than in osteoblastic lesions (median SUV_max_ 2.70), as active bone resorption releases TGF-β and IL-1β, which in turn upregulate FAP expression in CAFs and create a positive feedback loop that sustains osteolytic progression [23–26]. Conversely, the lower FAPI uptake in osteoblastic lesions likely reflects less active CAF infiltration and the physical barrier of dense sclerotic matrix. Collectively, these findings suggest that FAPI imaging provides not only diagnostic information but also biological insights into the dominant pathological process within the bone microenvironment, which may inform subtype-specific therapeutic strategies.

With the advent of effective treatment options, accurately detecting and monitoring responses in BM has become increasingly important. However, conventional imaging techniques such as CT, MRI, and BS are often recognized as being insensitive and nonspecific for response monitoring within clinically relevant timeframes [3]. While RECIST 1.1 remains the standard for evaluating solid tumors, its application to BM is constrained by inherent limitations [27]. Specialized criteria such as the MD Anderson (MDA) criteria have been proposed for bone-dominant metastases, its reliance on subjective interpretation introduces interobserver variability and limits standardization [28]. Our findings suggest that the uptake of FAPI in bone metastasis lesions correlates well with the conclusions drawn from the RECIST 1.1 criteria, though it is still preliminary and exploratory. This indicates that FAPI imaging may offer improvements in the assessment of therapeutic response in bone metastases compared to standard imaging modalities by quantifying biological processes related to both the bone microenvironment and tumor cells. However, the number of cases involved in this study is too small for definitive conclusions or for establishing an optimal delta-SUV_max_ cutoff, further investigation is warranted to establish the optimal cutoff values using larger datasets, and a prospective, large-cohort validation study is currently being designed to establish standardized criteria for FAPI-based treatment monitoring.

While PET/CT provides superior spatial resolution and image quality, the fundamental biological mechanism-FAP-targeted imaging-remains identical. Actually, our results are consistent with previous PET-based FAPI studies. Wu et al. and Guo et al. demonstrated that [^68^Ga]Ga-FAPI-04 PET/CT detects more bone metastases than [^18^F]FDG PET/CT, with higher SUV_max_ values and superior lesion-to-background contrast [29, 30]. A recent meta-analysis corroborated these findings, reporting pooled detection rates of 0.99 (patient-based) and 1.00 (lesion-based) for [^68^Ga]FAPI-04 [31]. This advantage appears to be consistent across bone metastasis subtypes, including both osteolytic and osteoblastic lesions [29, 32]. As expected, SUV_max_ values from SPECT-based [^99m^Tc]Tc-H7ND are not directly comparable to those from PET-based FAPI studies, owing to inherent physical differences between the two modalities. Nevertheless, the diagnostic performance, with a sensitivity of 96.9%, remained high. Beyond diagnostic accuracy, our study further extends these findings by evaluating the clinical impact of FAPI imaging on treatment decision-making and therapeutic response monitoring. This suggests that SPECT-based FAPI imaging, with its broader availability and lower cost, may serve as a practical alternative to PET-based FAPI in clinical settings where PET/CT access is limited.

Several limitations should be acknowledged. First, histopathological confirmation was not available for all patients, so lesion characterization primarily relied on imaging and clinical follow-up, which may introduce diagnostic uncertainties. Second, imaging examinations were not performed within a strictly identical time window for all patients, and disease progression during this interval may have affected cross-modality comparisons—a limitation inherent to the retrospective design. Future prospective studies with strictly standardized, same-week imaging protocols are necessary to eliminate this confounding factor. Third, the retrospective design and small sample sizes within tumor histology subgroups limited statistical power, thus larger multi-center studies are needed to evaluate tumor-specific differences. Finally, FAPI image interpretation was not performed independently of other imaging findings, which could have introduced ascertainment bias, although this reflects real-world clinical practice. Despite these limitations, this investigation provides exploratory evidence supporting the diagnostic utility of FAPI imaging for detecting BM across various malignancies, and multi-center prospective validation is needed.

## Conclusion

FAPI imaging with the [^99m^Tc]Tc-H7ND tracer demonstrated significantly higher diagnostic accuracy for BM compared to conventional imaging modalities, showing particular efficacy in identifying osteolytic lesions. Beyond diagnostic superiority, this technique demonstrates potential for improved clinical staging and theragnostic evaluation, with particular relevance for optimizing BM management.

### Methods

#### Patients

This retrospective study evaluates the diagnostic efficiency of FAPI imaging for various tumor types associated with BM, utilizing data collected from a registry (ChiCTR2100048093, Registration Date: June 30, 2021), approved by the First Affiliated Hospital of Xi’an Jiaotong University (Ethic approved No.: XJTU1AF2021LSK-021). The study is reported in accordance with the Standards for Reporting of Diagnostic Accuracy (STARD) guidelines. Between June 2021 and September 2023, consecutive patients with various malignancies were recruited for FAPI imaging based on predefined eligibility criteria (detailed in the Supplementary Material) after providing informed consent.

Consecutive patients with “suspected bone metastases” were enrolled, based on the following criteria: (1) patients with confirmed or suspected BM based on prior imaging modalities; (2) patients with “positive” bone lesions identified on FAPI imaging, regardless of whether the lesions were benign or malignant; (3) patients with high risk of bone metastases (high clinical suspicion of bone metastases based on a history of localised pain or tenderness over a bone area; with either elevated serum alkaline phosphatase (ALP) or tumor markers but cannot be explained by extraskeletal metastases). To avoid incorporation bias, FAPI findings were not used as the sole incorporation criterion. Patients were excluded if they lacked regular follow-up or review results or were unable to adhere to the prescribed treatment protocol following FAPI imaging. Meanwhile, other conventional imaging data concurrent with FAPI imaging (primarily within 28 days) were collected, including CT, MRI, BS, and/or [^18^F]FDG PET/CT. For patients undergoing multiple FAPI follow-up examinations, data collected during the initial FAPI imaging session were statistically analyzed.

### Preclinical experiments and radiopharmaceutical preparation

The preclinical experiments of FAPI agent, [^99m^Tc]Tc-H7ND (with structure showing in Supplementary Fig. S4), was summarized in Supplementary materials. Briefly, quality control was performed by assessing radiochemical purity (RCP), with values consistently above 95% for preclinical and clinical use (Supplementary Fig. S5). In vitro cell binding assays were conducted to evaluate the affinity of [^99m^Tc]Tc-H7ND for FAP (Supplementary Fig. S6). Pharmacokinetic properties in mice were assessed by measuring the RCP of [^99m^Tc]Tc-H7ND in urine and whole-body radioactivity over time (Supplementary Fig. S7). Tumor-specific targeting and imaging capabilities of [^99m^Tc]Tc-H7ND were validated using micro-SPECT/CT (Supplementary Fig. S8). Biodistribution in mice was evaluated by calculating the percentage of injected dose per gram of tissue (%ID/g) at 1 h and 4 h post-injection (Supplementary Fig. S9). The reaction mixture was filtered through a 0.22-µm sterile filter before injection.

### Imaging protocol and reconstruction

The [^99m^Tc]Tc-H7ND was administered intravenously at a dose of 12.95 MBq/kg (0.35 mCi/kg) (± 10%), with individual doses ranging from 629 to 999 (17 to 27 mCi). Dynamic biodistribution was evaluated via whole-body planar scintigraphy at 10, 30, 60, 120, and 240 min post-injection in four representative patients as previously described [33]. Regional (neck-to-pelvis) SPECT/CT fusion imaging was performed in all patients 45–60 min post-injection using a Discovery NM/CT 670Pro system (GE Healthcare, USA) equipped with low-energy, high-resolution collimators. Low-dose CT was acquired for attenuation correction and anatomical localization. Image reconstruction followed the protocol detailed in reference [33]. Patients were monitored for adverse effects, with self-reported symptoms documented 30 min post-examination, and followed up by telephone within 24 to 48 h. The biodistribution and dosimetric parameters of FAPI agent [^99m^Tc]Tc-H7ND is summarized in Supplementary Fig. S10 and Table S3.

### Imaging review

Three board-certified nuclear medicine physicians independently evaluated the FAPI PET/CT scans, resolving discrepancies through consensus. Regions of interest (ROIs) were manually delineated on transaxial slices to encompass areas of visually elevated tracer uptake. Standardized uptake values (SUVs) were calculated using a validated algorithm [34]. Background bone activity were determined by placing 2-cm diameter ROIs on adjacent normal-appearing bone tissue, yielding SUV_mean_ of bone (SUV_bone_). Maximum SUV ratios (SUVR) were derived by dividing the Maximum SUV (SUV_max_) of the bone metastatic lesion by the corresponding SUV_bone_. Regions exhibiting higher uptake than normal bone tissue, after excluding physiological uptake and traumatic fracture, were deemed “positive bone lesions”.

Bone metastases (BM) were categorized as osteolytic, osteoblastic, or mixed types. For each patient, the three lesions with the highest FAPI uptake for each type were recorded. In patients with multiple scans, up to five lesions—with no more than two per type—were selected to assess changes in SUV_max_ (delta-SUV_max_). To account for lesion distribution, metastatic sites were categorized into the following anatomical regions: spine, ribs, pelvis, limbs, and skull. The number of lesions per site was classified as 0, 1, 2, 3, or ≥ 4 (more than 3).

### Diagnosis and follow-up

This section closely adheres to the methodology established in our prior investigation [33], with supplementary guidelines formulated for the evaluation of bone metastasis (BM) diagnosis, as described in an earlier study [35]. Briefly, the reference standard for BM diagnosis comprised the following: Histopathology (biopsy or surgery) served as the gold standard when available. For cases without pathological confirmation: (1) the lesion showed typical features of bone metastasis (osteolytic, osteoblastic, or mixed changes) on at least two conventional imaging modalities, based on findings indicative of BM [5; 14]; (2) follow-up imaging (CT, MRI, or BS) performed at least 6 months after the initial scan demonstrated unequivocal enlargement, new lesions, or post-treatment sclerosis consistent with metastatic involvement. All imaging reviews were performed independently by three experienced nuclear medicine physicians, who were not blinded to FAPI results due to the retrospective design; however, the reference standard assessment was performed separately and did not rely solely on FAPI findings. Unverifiable lesions were excluded, and suspected lesions were monitored for a minimum of 6 months. All findings and any changes were interpreted through a consensus approach. For patients in whom a change in clinical management was indicated, adjustments in oncologic management were based on decisions made by the Multidisciplinary Treatment (MDT) group.

### Statistical analyses

All statistical analyses were performed using SPSS version 27.0 statistical analysis software (IBM, Armonk, NY, USA). Organ biodistribution was assessed using the percentage of initial injected activity after decay correction per-organ (%ID). Descriptive statistics were utilized to calculate the number of metastases and SUV values, summarized as mean ± SD or median (IQR) based on the data distribution. The Mann-Whitney U test was used for two-group independent comparisons, and the Wilcoxon signed-rank test was used for paired comparisons. The McNemar test were employed to compare the diagnostic performance between FAPI imaging and other examinations concerning the presence of BM on a per-patient basis, using the reference standard. A two-tailed *p* value of < 0.05 was considered statistically significant.

## Supplementary Information

Supplementary Material 1

## Data Availability

The data that support the findings of this study are available from the corresponding author upon reasonable request.

## Abbreviations

BM: Bone metastases
BS: Bone scintigraphy
CAF: Cancer-associated fibroblast
FAPI: Fibroblast activation protein inhibitor
IQR: Interquartile range
SUV: Standard uptake value
SUVR: Standard uptake value ratio
