# Supplementary material for: Retrospective study of FAPI imaging in bone metastases: diagnostic utility and therapeutic relevance across cancer types in a single-center real-world cohort
Source: EJNMMI Res. 2026 Aug 11;16:156. doi: 10.1186/s13550-026-01502-z (PMC13745607; doi:10.1186/s13550-026-01502-z)
Supplement: Supplementary file 1 [file 13550_2026_1502_MOESM1_ESM.docx]

**Supplementary material for**

**Retrospective Study of FAPI Imaging in Bone Metastases: Diagnostic Utility and Therapeutic Relevance Across Cancer Types in a Single-center Real-world Cohort**

**Supplementary Methods**

**Materials and methods for synthesis of [^99m^Tc]Tc-H7ND with quality control**

All reagents, unless otherwise specified, were of analytical grade and commercially available. High-quality Milli-Q water (18.2 MΩ/cm, Milipore, USA) was used for preparing solutions. Hydrazinonicotinamide-7ND (HYNIC-7ND, abbreviated as H7ND) radiolabeling kit were provided by Pharbers Genesis Pharmaceutical Technology Co., Ltd. Na^99m^TcO_4_ was obtained from a commercial ^99^Mo/^99m^Tc generator (Beijing Atom High Tech Co., Ltd.). For synthesis of [^99m^Tc]Tc-H7ND, 1 mL of 740-1,110 MBq (20-35 mCi) Na^99m^TcO4 saline solution was added to a HYNIC-7ND kit and incubated at 100°C for 30 minutes. After cooling, the radiochemical purity (RCP) was determined by instant thin layer chromatography (iTLC, **Fig. S5**) using an iTLC Scanner AR-2000 (Bioscan, USA) and glass microfiber chromatography paper impregnated with silica gel (iTLC-SG, Agilent Technologies, USA), and was consistently above 95% for clinical use. Briefly, the reaction mixture was filtered through a 0.22-μm sterile filter before injection. Two types of eluants were used in iTLC analysis. When acetone is used as the eluant, ^99m^TcO_4_^-^ migrated to the solvent front, while ^99m^Tc-colloid and ^99m^Tc complexes remained at the origin. When a 1:1 mixture of acetonitrile and saline is used as the eluant, ^99m^Tc complexes and ^99m^TcO_4_^-^ migrated to the solvent front, while ^99m^Tc-colloid remained at the origin.

**In Vitro Cell Binding Assay of [^99m^Tc]Tc-H7ND**

For the cell binding assay, HT-1080-hFAP cells stably transfected with hFAP were used as positive controls, while HT-1080 cells served as negative controls. Briefly, culture media containing HT-1080-FAP and HT-1080 cells were centrifuged at 150 ×g for five minutes. The cells were then resuspended and washed twice with 10 mL PBS, and collected into 1.5 mL EP tubes at a density of 2×10^6 cells per tube and blocked with 2% BSA solution at room temperature for 30 minutes. ^99m^Tc-labeled H7ND was added to the cells at a concentration of 11 kBq per tube, followed by incubation on ice for 20 minutes. To study the inhibition of probe-cell interaction, a 1000-fold molar excess of H7ND was added to the corresponding EP tubes and co-incubated. After incubation, cells were resuspended in cold PBST and centrifuged at 150 ×g for five minutes, then washing five times. Finally, the washed cells were lysed with 2 mol/L NaOH, and the lysates were collected and measured for radioactivity using a γ-counter. Each experimental group included four replicates. The binding values were expressed as the percentage of added dose (%AD) of radioactivity counts per minute, corrected of attenuation.

**In Vivo Stability and Metabolism of [^99m^Tc]Tc-H7ND in Normal BALB/c Mice**

BALB/c mice (n=6) were divided into two groups (3 mice/group). Each group received an intravenous injection of [^99m^Tc]Tc-H7ND (100 μL, ~37 MBq). Urine samples were collected at 0.5 and 1 h post-injection, and the radiochemical purity of [^99m^Tc]Tc-H7ND in urine was analyzed using radio-high-performance liquid chromatography (radio-HPLC). Whole-body radioactivity was measured at 0, 0.5, 1, 2, and 4 h post-injection under physiological conditions.

**SPECT/CT Imaging of [^99m^Tc]Tc-H7ND in U87MG Tumor-Bearing Mice**

The prepared [^99m^Tc]Tc-H7ND was diluted to 37 MBq/100 μL, and each U87MG tumor-bearing mouse was intravenously injected with 100 μL (37 MBq) via the tail vein. SPECT/CT imaging was performed at 0.5, 1, 2, 4, 8, 12, and 24 hours post-injection. For the blocking group, mice were co-injected with 100 μL (500 μg) of unlabeled H7ND alongside the imaging agent and imaged at 0.5 hours post-administration. During imaging, mice were anesthetized with a 1.5% isoflurane-oxygen mixture. SPECT images were reconstructed and fused with CT images to generate 3D tomographic views. Posterior projections are presented, with tumor locations indicated by arrows.

**Biodistribution of [^99m^Tc]Tc-H7ND in U87MG Tumor-Bearing Mouse Models**

Eight BALB/c nude mice (6 weeks old) bearing U87MG xenografts were randomly divided into two groups (four mice per group). [^99m^Tc]Tc-H7ND was dissolved in saline (370 kBq/100 μL), and 100 μL (370 kBq) was administered via tail vein injection. Animals were euthanized at 1 and 4 h post-injection. Blood and major organs were collected, weighed, and measured for radioactive counts per minute (cpm). After decay correction, the percentage of injected dose per gram of tissue (%ID/g) was calculated. Biodistribution data are presented as mean ± standard deviation (mean ± SD, n=4).

**Eligibility criteria for FAPI imaging with [^99m^Tc]Tc-H7ND**

Inclusion criteria: (a) Subjects with newly diagnosed or previous treated malignant cancer; (b) The expected survival was more than 12 weeks; (c) Understand and sign informed consent voluntarily with good compliance.

Exclusion criteria: (a) Pathologically confirmed non-malignant tumor; (b) The function of liver and kidney was seriously abnormal; (c) Preparation for pragnant, pregnant and lactating women; (d) Inability to lie flat for half an hour;(e) Suffering from claustrophobia or other mental disorders; (f) Other researchers considered it unsuitable to participate in the trial.

**Supplementary figures**

**Fig. S1** Number of bone metastatic lesions detected by various imaging modalities across all observed patients in patient-based analysis. BS, Bone scintigraphy; FDG: [^18^F]FDG PET/CT. ns, none significance; *, *p* <0.05; ***, *p* <0.001.

**Fig. S2** Representative FAPI imaging in a patient presenting with unexplained bone pain. **a**, Maximum Intensity Projection (MIP) of FAPI imaging. CT and SPECT/CT fusion figure of bone lesion (**b**) and stomach lesion (**c**) were confirmed by pathological HE stains with 100× magnification (**d** and **e**, respectively). White arrows indicated corresponding HE stain slice.

**Fig. S3** Comparison of the results from patients undergoing reviewed FAPI imaging. **a**, The relationship between changes of the SUV_max_ (delta-SUV_max_) of bone lesions and the RECIST 1.1 evaluation in 8 reviewed patients. nPR/nPD: non-partial response/non-progressive disease; SD: stable disease; PD: progressive disease. **b**, Discovery of new bone lesions in a patient's initial FAPI imaging, together with liver metastases in both FAPI imaging (above) and contrast-enhanced CT (ceCT, top right of the above); while, treatment strategy was not changed and 2 months later the metastatic lesions were progression with more lesions and higher uptake of FAPI to be found (below), with the contemporaneous ceCT shown in the top right of the below.

**Fig. S4** Structure of [^99m^Tc]Tc-H7ND.

**Fig. S5** The iTLC-SG analysis of [^99m^Tc]Tc-H7ND after radiolabeling. **a**, acetone as eluant. **b**, 1:1 mixture of acetonitrile and saline as eluant.

**Fig. S6** In vitro cell binding assay. ***, *p* < 0.001.

**Fig. S7** In vivo metabolism of [^99m^Tc]Tc-H7ND in healthy mice. **a,** radiochemical purity of ^99m^Tc-H7ND in urine was analyzed using radio-HPLC at 0.5 and 1 h post-injection (p.i.). **b,** Whole-body radioactivity was measured at 0, 0.5, 1, 2, and 4 h p.i. under physiological conditions.

**Fig. S8** SPECT/CT Imaging of [^99m^Tc]Tc-H7ND in U87MG Tumor-Bearing Mice. White arrow represented the xenograft tumor.

**Fig. S9** Biodistribution results of [^99m^Tc]Tc-H7ND in the U87MG tumor-bearing mice model. **a**, the percentage of injected dose per gram of tissue (%ID/g) was calculated at 1 h and 4 h p.i.. **b,** Ratio of %ID/g between tumor (T) to target organs (B: blood; K: kidney; L: liver; M: muscle) at 1 h and 4 h p.i..

**Fig. S10** Dynamic bio-distribution of [^99m^Tc]Tc-H7ND in different vital organs over time. **a**, percentage of Injection Dose (%ID)/organ of [^99m^Tc]Tc-H7ND in heart, liver, lungs, kidneys, pancreas, spleen, brain, bone marrow, thyroid, parotid glands and submandibular glands in different times. Data represent mean ± SD (n=4). **b**, example of background in one patient on planar scintigraphy images: whole body ^99m^Tc-H7ND scintigraphy was performed at 10, 30, 60, 120 and 240 min post-injection.

**Supplementary tables**

**Table S1** The Patient-based Diagnostic Value of Each Method for Detection of BM

| Basis of analysis and modality | Sensitivity  TPR (%) | Specificity  TNR (%) | Negative predict value  NPV (%) | Positive predict value  PPV (%) | Accuracy  ACC (%) | You-den’s index |
| --- | --- | --- | --- | --- | --- | --- |
| FAPI (n=80) | 96.9 (62/64) | 93.8 (15/16) | 88.2  (15/17) | 98.4 (62/63) | 96.3 (77/80) | 0.907 |
| 95% CI | 88.2-99.5 | 67.7-99.7 | 62.3-97.9 | 90.3-99.9 | 89.4-99.2 |  |
| CT　(n=73) | 59.7 (34/57) | 93.8 (15/16) | 39.5  (15/38) | 97.1 (34/35) | 67.1  (49/73) | 0.535 |
| 95% CI | 45.8-72.4 | 69.8-99.8 | 24.0-56.6 | 85.1-99.9 | 55.1-77.7 |  |
| MRI　(n=24) | 76.2 (16/21) | 100  (3/3) | 37.5  (3/8) | 100  (16/16) | 79.1  (19/24) | 0.762 |
| 95% CI | 52.8-91.8 | 29.2-100 | 8.5-75.5 | 79.4-100 | 57.9-92.9 |  |
| BS　(n=36) | 86.6 (26/30) | 83.3  (5/6) | 55.6  (5/9) | 96.3 (26/27) | 86.1 (31/36) | 0.699 |
| 95% CI | 68.3-95.6 | 36.5-99.1 | 22.7-84.7 | 79.1-99.8 | 70.5-95.3 |  |
| FDG  (n=14) | 66.7  (8/12) | 100  (2/2) | 66.7  (4/6) | 100  (8/8) | 71.4 (10/14) | 0.667 |
| 95% CI | 34.9-90.1 | 15.8-100 | 22.3-95.7 | 63.1-100 | 41.9-91.6 |  |
| Abbreviation: BM: bone metastases; BS, Bone scintigraphy; FDG: [^18^F]FDG PET/CT; TPR: true positive rate; TNR: true negative rate | | | | | | |

**Table S2** Lesion-based changes of the uptake of BM who underwent reviewed FAPI imaging

| No./Sex/Age | Primary tumor | RECIST 1.1 evaluation | Type of BM | First-time SUV_max_ | Changes of treatment regimen | Reviewed SUV_max_ | delta-SUV_max_ |
| --- | --- | --- | --- | --- | --- | --- | --- |
| 1/M/52 | Prostate Cancer | SD | Mixed | 9.73 | Yes | 13.68 | 40.60% |
|  |  |  | osteoblastic | 11.97 |  | 7.62 | -36.34% |
|  |  |  | Mixed | 10.54 |  | 11.27 | 6.93% |
|  |  |  | osteoblastic | 10.4 |  | 7.66 | -26.35% |
|  |  |  | Mixed | 12.69 |  | 8.53 | -32.78% |
| 2/M/66 | Lung adenocarcinoma | SD | Mixed | 6.25 | No | 2.9 | -53.60% |
|  |  |  | osteoblastic | 1.95 |  | 1.1 | -43.59% |
|  |  |  | osteoblastic | 0.94 |  | 0.61 | -35.11% |
| 3/M/60 | Lung squamous carcinoma | SD | osteolytic | 12.82 | No | 9.1 | -29.02% |
|  |  |  | Mixed | 7.06 |  | 5.89 | -16.57% |
| 4/M/63 | Bladder cancer | nPR/nPD | Osteoblastic | 6.13 | Yes | 2.41 | -60.69% |
|  |  |  | Osteoblastic | 3.92 |  | 1.52 | -61.22% |
| 5/F/53 | Lung adenocarcinoma | nPR/nPD | Osteoblastic | 11.99 | No | 2.69 | -77.56% |
|  |  |  | Osteoblastic | 8.29 |  | 7.3 | -11.94% |
|  |  |  | Osteoblastic | 5.55 |  | 2.75 | -50.45% |
| 6/M/30 | Thymic carcinoma | nPR/nPD | osteolytic | 10.62 | Yes | 1.69 | -84.09% |
|  |  |  | Mixed | 11.18 |  | 3.88 | -65.30% |
|  |  |  | Mixed | 11.5 |  | 1.78 | -84.52% |
|  |  |  | Mixed | 9.77 |  | 1.55 | -84.14% |
|  |  |  | osteolytic | 9.27 |  | 2.31 | -75.08% |
| 7/M/55 | Small cell lung cancer | PD | Mixed | 10.78 | Yes | 13.54 | 25.60% |
|  |  |  | osteolytic | 6.89 |  | 11.24 | 63.13% |
|  |  |  | Mixed | 5.15 |  | 8.54 | 65.83% |
|  |  |  | Mixed | 4.14 |  | 5.47 | 32.13% |
| 8/F/59 | Lung adenocarcinoma | PD | Mixed | 17.83 | Yes | 22.56 | 26.53% |
|  |  |  | osteolytic | 17.91 |  | 32.01 | 78.73% |
|  |  |  | osteoblastic | 7.92 |  | 15.39 | 94.32% |
|  |  |  | osteoblastic | 4.83 |  | 10.87 | 125.05% |
|  |  |  | osteoblastic | 7.8 |  | 23.14 | 196.67% |

Abbreviation: SD: stable disease; nPR/nPD: non-partial response/non-progressive disease; PD: progressive disease

**Table S3** Dosimetric data of [^99m^Tc]Tc-H7ND

| Absorbed dose of target organs  (mSv/MBq) | [^99m^Tc]Tc-H7ND (n=4) | |
| --- | --- | --- |
|  | Mean | SD |
| Adrenals | 4.07E-04 | 4.30E-05 |
| Brain | 7.95E-05 | 4.18E-05 |
| Breasts | 1.44E-04 | 1.59E-05 |
| Gallbladder Wall | 2.96E-04 | 2.93E-05 |
| LLI Wall | 4.56E-05 | 6.03E-06 |
| Small Intestine | 1.00E-04 | 6.58E-06 |
| Stomach Wall | 2.25E-04 | 3.45E-05 |
| ULI Wall | 1.10E-04 | 8.33E-06 |
| Heart Wall | 2.85E-03 | 5.34E-04 |
| Kidneys | 2.79E-03 | 2.48E-04 |
| Liver | 6.44E-04 | 5.68E-05 |
| Lungs | 1.11E-03 | 9.13E-05 |
| Muscle | 9.64E-05 | 8.80E-06 |
| Ovaries | 5.47E-05 | 6.35E-06 |
| Pancreas | 1.32E-03 | 3.76E-04 |
| Red Marrow | 3.65E-04 | 5.65E-05 |
| Osteogenic Cells | 2.89E-04 | 3.30E-05 |
| Skin | 4.06E-05 | 3.45E-06 |
| Spleen | 7.47E-04 | 2.12E-04 |
| Testes | 5.62E-06 | 9.53E-07 |
| Thymus | 2.69E-04 | 3.75E-05 |
| Thyroid | 2.46E-03 | 5.98E-04 |
| Urinary Bladder Wall | 2.00E-05 | 2.80E-06 |
| Uterus | 4.36E-05 | 4.50E-06 |
| Total Body | 1.64E-04 | 1.50E-05 |
|  |  |  |
| Effective Dose Equivalent (mSv/MBq) | 7.96E-04 | 1.01E-04 |
| Effective Dose (mSv/MBq) | 4.34E-04 | 4.15E-05 |
